# A Novel Allosteric Inhibitor Targeting IMPDH at Y233 Overcomes Resistance to Tyrosine Kinase Inhibitors in Lymphoma

**DOI:** 10.3390/cancers17203389

**Published:** 2025-10-21

**Authors:** Nagarajan Pattabiraman, Cosimo Lobello, David Rushmore, Luca Mologni, Mariusz Wasik, Johnvesly Basappa

**Affiliations:** 1 Veracure Biosciences Inc., Silver Spring, MD 20901, USA; pattabiraman@veracurebiosciences.com; 2 Department of Pathology, Fox Chase Cancer Center, Philadelphia, PA 19111, USA; 3 Department of Medicine and Surgery, University of Milano-Bicocca, 20126 Monza, Italy

**Keywords:** *IMPDH2*, PI3P, tyrosine phosphorylation, in silico screening, IMPDH drug discovery

## Abstract

Background/Objective: Oncogenic tyrosine kinases (TKs) such as ALK and SRC promote cancer progression, but their effects on metabolic enzymes are still not well understood. This study examines how TK signaling regulates inosine monophosphate dehydrogenase 2 (IMPDH2), a rate-limiting enzyme in purine biosynthesis, and assesses its potential as a therapeutic target. Methods: Phosphoproteomic screening and in vitro kinase assays were used to identify phosphorylation sites on IMPDH2. Lipid-binding assays explored the role of phosphatidylinositol 3-phosphate (PI3P) in IMPDH2 regulation. Structure-based virtual screening discovered small-molecule allosteric inhibitors, which were tested in lymphoma cell models, including ALK and BTK-inhibitor resistant lines. Results: Here, we identify Inosine monophosphate dehydrogenase-2 (IMPDH2), a rate-limiting enzyme in purine biosynthesis, as a novel substrate of ALK and SRC. We show that phosphorylation at the conserved Y233 residue within the allosteric domain enhances IMPDH2 activity, linking TK signaling to metabolic reprogramming in cancer cells. We further identify PI3P as a natural lipid inhibitor that binds IMPDH2 and suppresses its enzymatic function. Using structure-based virtual screening, we developed Comp-10, a first-in-class allosteric IMPDH inhibitor. Unlike classical active-site inhibitors such as mycophenolic acid (MPA), Comp-10 decreases IMPDH1/2 protein levels, blocks filament (rod/ring) formation, and inhibits the growth of ALK and BTK inhibitor-resistant lymphoma cells. Comp-10 acts post-transcriptionally and avoids compensatory IMPDH upregulation observed with MPA (rod/ring) formation, and inhibited growth in TKI-resistant lymphoma cells. Notably, Comp-10 avoided the compensatory IMPDH upregulation observed with MPA. Conclusion: These findings uncover a novel TK–IMPDH2 signaling axis and provide mechanistic and therapeutic insight into the allosteric regulation of IMPDH2. Comp-10 represents a promising therapeutic candidate for targeting metabolic vulnerabilities in tyrosine kinase driven cancers.

## Simple Summary

Cancer cells often rely on altered metabolism to support their rapid growth. We discovered that two cancer-driving proteins, ALK and SRC, directly modify and activate a key metabolic enzyme called IMPDH2. This modification occurs at a specific site (Y233) and boosts the enzyme’s activity, helping cancer cells make the building blocks of DNA. We also found that a natural lipid molecule, PI3P, can bind to and inhibit IMPDH2. Based on this knowledge, we developed a new drug candidate, Comp-10, which targets the regulatory region of IMPDH2. Unlike existing drugs, Comp-10 reduces IMPDH2 protein levels, blocks its activity, and prevents the formation of harmful enzyme structures in cancer cells. Importantly, it works in cancer cells that are resistant to current treatments. These findings suggest that targeting IMPDH2 in this new way could lead to better therapies for cancers driven by ALK, SRC, or similar proteins.

## 1. Introduction

Humans and other mammals express two IMPDH isoforms, IMPDH1 and IMPDH2, each comprising 514 amino acids and sharing 84% sequence identity. Both isoforms contain a catalytic domain that binds substrates and a regulatory Bateman domain, which modulates enzymatic activity through allosteric interactions with the catalytic core [1]. The IMPDH regulatory (Bateman) domain contains ATP- and GTP-binding sites. IMPDH exists as a constitutive tetramer, and nucleotide binding promotes reversible dimerization of the regulatory domains, leading to octamer formation [2]. IMPDH1 is constitutively expressed in normal lymphocytes but is highly upregulated in a subset of small cell lung cancers (SCLC) [3]. IMPDH2 is overexpressed in hematological malignancies, including human leukemic cell lines and BCR-ABL-positive acute myelogenous leukemia [4], in chronic myelogenous leukemia [5] and other cancers, such as triple-negative breast cancer [6], prostate cancer [7,8], kidney cancer [9], nasopharyngeal carcinoma [10], in a subset of small-cell lung cancers [3,11], in non-small cell lung cancer [12] and in glioblastoma [13,14] and brain metastases [15]. Proteomic profiling of colorectal cancer plasma identified IMPDH2 as a potential biomarker [16]. Whereas protein tyrosine phosphorylation makes up only 2.5%, it has significant effects on nearly every aspect of cellular physiology [17]. Our previous work demonstrated that ALK and SRC kinase-mediated tyrosine phosphorylation of ATP-citrate lyase (ACLY) regulates its function [18,19]. Although IMPDH2 overexpression in solid tumors is well documented, its post-translational regulation, particularly by tyrosine phosphorylation remains poorly understood. Here, we report for the first time that IMPDH2 is phosphorylated on a critical tyrosine residue by oncogenic kinases, as demonstrated through in vitro kinase assays and mass spectrometry-based phosphoproteomic analysis.

IMPDH1 and IMPDH2 pose a major challenge for isoform-specific drug development due to their 84% sequence identity. To overcome this, we analyzed sequence differences and hypothesized that the two isoforms may possess distinct phosphoinositide (PI) binding sites. PIs are lipid second messengers critical for membrane trafficking, metabolism, growth, signaling, and autophagy. Their phosphorylation generates seven distinct species, including PI3P, a key marker of endosomal and autophagic membranes. PI3P is recognized by FYVE and PX domain-containing proteins, suggesting that isoform-specific PI interactions could be leveraged for selective targeting of IMPDH isoforms [20,21]. FDA-approved IMPDH inhibitors—such as Mycophenolic acid (MPA), Mycophenolate mofetil (MMF), Ribavirin, and Mizoribine target the catalytic domains of both IMPDH1 and IMPDH2 and are used clinically for immunosuppressive and antiviral therapy [22]. These inhibitors also promote the formation of Rods and Rings (RRs), or IMPDH filaments, which are non-membrane-bound intracellular polymeric structures [23,24,25].

This study shows that mycophenolic acid (MPA) induces the expression of catalytically inactive IMPDH2 and promotes filament formation in T-cell and B-cell lymphomas. This filamentous assembly may contribute to the toxicity and off-target effects of current IMPDH inhibitors, highlighting the need for more selective therapeutics. We identify IMPDH2 as a direct substrate of the oncogenic kinases ALK and SRC and demonstrate that PI3P binding specifically inhibits IMPDH2 activity but not that of IMPDH1. Based on these findings and structural modeling, we performed silico screening and discovered a new allosteric inhibitor, comp-10. This inhibitor specifically targets the allosteric domain and is distinct from current IMPDH inhibitors, opening new possibilities for future therapeutic development.

## 2. Materials and Methods

### 2.1. Reagents and Antibodies

Recombinant human IMPDH1 (Catalog # 8904-DH) and IMPDH2 (catalog# Catalog # 8349-DH) proteins were obtained from R&D Systems, Minneapolis, MN, USA. IMPDH activity assay kit was from Biovision (Catalog#K495) or abcam (catalog#ab283395), Waltham, MA, USA. PIP Strips—Lipid-Protein Interaction Assay (catalog#P-6001), PIP Arrays—Lipid-Protein Interaction Assay (catalog# P-6001), PI(3)P Beads (catalog#P-B003A), PI(4,5)P2 diC4 (catalog#P-4504) and PI(3)P diC8 (catalog#P-3008A) were purchased from Echelon Biosciences, Salt Lake City, UT, USA. All other reagents are from Sigma-Aldrich, Saint Louis, MO, USA. Cell Proliferation Reagent WST-1 (Millepore-sigma), CellTiter 96^®^ Non-Radioactive Cell Proliferation Assay (MTT) kit (catalog#G4001) from Promega, Madison, WI, USA. HA Tag Monoclonal Antibody (Cat#26183, RRID: AB_10978021), Pierce IP Lysis Buffer (Cat#87787), Pierce Anti-HA Magnetic Beads (Cat# 88837, RRID: AB_2861399) Halt Protease and Phosphatase Inhibitor (Cat#78440), ALK Recombinant Human Protein (Cat# PV3867) and SRC Recombinant Human Protein (Cat# P3044) all of these were from Thermo Fisher Scientific, Waltham, MA, USA. Mizoribine (Cat #S1384), Ribavarin (Cat #S2504), mycophenolic acid (Cat# S2487), Mycophenolate mofetil (Cat#S1501), certinib (Cat#S7083). All these small-molecule inhibitors were purchased from SelleckChem, Houston, TX, USA. IMPDH1 (RRID:AB_2878992, Cat# 22092-1-AP), IMPDH2 (RRID:AB_2127351, Cat# 12948-1-AP) and GAPDH (RRID:AB_2107436, Cat# 60004-1-Ig) from Proteintech, Rosemont, IL, USA. HA-Tag (C29F4) Rabbit (RRID:AB_1549585, Cat# 3724S), Phospho-ALK (Tyr1604) Antibody (RRID:AB_331047, Cat# 3341S), ALK (D5F3^®^) XP^®^ Rabbit (RRID:AB_11127207, Cat# 3633S) from Cell Signaling Technology, Danvers, MA, USA.

### 2.2. Plasmids and Lentivirus Products

Custom Plasmid Preparation: IMPDH2-HA tagged_pLenti_MS2-P65-HSF1_mCherry, synthesized at GenScript, Piscataway, NJ. pLenti-EGFP-2xFYVE (Plasmid #136996, pLenti-EGFP-2xFYVE was a gift from Ken-Ichi Takemaru (Addgene plasmid Cat#136996; http://n2t.net/addgene:136996; RRID:Addgene_136996 accessed on 11 March 2022), 3rd Gen. Packaging Mix & Lentifectin Combo Pack from Applied Biological System, Richmond, BC V6V 2J5, Canada (ABM good# LV053-G074), Lenti-X™ Concentrator from TAKARA, San Jose, CA, USA (catalog# 631231).

### 2.3. MCL, DLBCL, and ALCL Cell Lines

MCL and MCL-RL cells were derived from a patient with MCL at the University of Pennsylvania, Philadelphia, PA. JeKo-1, Maver Rec-1, Granta519, DLBCL, OCI-LY1, OCI-LY4, OCI-LY8, and TOLEDO; SUDHL-1, JB6, Karpas 299, SUP-M2, L82, and SR786 cell lines were derived from ALK+ALCL patients and cultured as described in earlier [26]. Human CD4+ cells transduced with NPM-ALK, NA1, were created by our group as described earlier [27]. The cell lines were regularly tested for Mycoplasma contamination using Mycoplasma detection kits from Thermo Fisher Scientific (Waltham, MA, USA) and were authenticated. Cells were maintained in RPMI-1640 medium supplemented with 10% fetal bovine serum (FBS) and 1% penicillin/streptomycin (Pen/Strep) in a humidified incubator at 37 °C with 5% CO_2_.

### 2.4. ALK Inhibitor (ALKi)-Resistant ALCL Cell Lines

ALKi crizotinib-resistant cell lines described earlier, KARPAS299 CR06 (resistant up to 600 nM of crizotinib and cultured at that concentration) and SUPM2 CR03 (resistant up to 300 nM of crizotinib and cultured at that concentration), and ALKi lorlatinib-resistant cell lines described earlier, KARPAS LR1000 (resistant up to 1000 nM of lorlatinib and cultured at 300 nM of lorlatinib) and SUPM2 LR1000 (resistant up to 1000 nM of lorlatinib and cultured at 300 nM of lorlatinib) all of these cell lines were obtained from Drs. Carlo Gambacorti-Passerini and Luca Mologni, University of Milano-Bicocca, Italy [28]. The cell lines were grown in RPMI medium supplemented with 10% FBS and 1% penicillin/streptomycin in a humidified incubator at 37 °C with 5% CO_2_.

### 2.5. IMPDH1 and IMPDH2 PIP Strip and PIP Array Binding Assay

For initial experiments, human recombinant IMPDH1 and IMPDH2 proteins were diluted to 1 µg/ml in 3% BSA and dissolved in PBS buffer. Next, the IMPDH2 concentration was further diluted to 100 ng/ml. The diluted proteins were subjected to a PIP strip lipid-protein interaction assay as described earlier. Based on IMPDH2’s unique binding to PI3P compared to IMPDH1, we further validated IMPDH2’s binding to PI3P on a PIP array coated with various concentrations.

### 2.6. IMPDH1 and IMPDH2 Activity Assay in the Presence of PIP2 (PI(4,5)P2) and PI3P (PI(3)P diC8)

Activity assays on human recombinant IMPDH1 and IMPDH2 were performed using a Synergy H1 microplate reader from BioTek, Santa Clara, CA, USA 96-well plate readers, and a commercially available kit from Biovision, Inc. (now part of Abcam, Waltham, MA, USA) According to the kit instructions, the phospholipids PIP2 and PI3P were diluted in activity buffer and preincubated with recombinant IMPDH1 or IMPDH2 at concentrations ranging from 50 µM to 200 µM for 15 min. After this incubation, a substrate solution was added to the wells to start enzyme activity. The activity was measured every minute for 30 to 60 min.

### 2.7. In Vitro Kinase Assay and LC-MS/MS Phosphoproteomics Analysis

We performed an in vitro kinase assay on human recombinant IMPDH2 in the presence of active anaplastic lymphoma kinase (ALK) and SRC kinase. We subjected it to LC-MS/MS analysis as we described in our previous study [18].

### 2.8. Structure-Based In Silico Screening of IMPDH Inhibitors

The National Cancer Institute (NCI) maintains a database of compounds, known as the Mechanistic Set VI, which comprises 811 compounds derived from the 37,836 open compounds tested in the NCI human tumor 60-cell line screen. This mechanistic diversity set was chosen to represent a broad range of growth inhibition patterns in the NCI60 cell line screen based on the GI50 activity of the compounds. Compounds tested in the NCI-60 cell line screen were clustered using the FASTCLUS procedure in the SAS 9.4 statistical package. This algorithm is based on MacQueen’s k-means algorithm, which minimizes the sum of squared distances from the cluster means. The procedure resulted in 1272 clusters. A single representative compound from each cluster, for which an adequate supply of material was available, was chosen. Some clusters are not represented in the set, as insufficient material was available. The database of compounds was downloaded. We subjected the compounds in the database with the following filter: (1) 100 ≤ Molecular Weight ≤ 500 (2) Only one molecule, (3) 2 ≤ Number of rotatable bonds ≤ 6 (4) Lipenski druglike = 1 and no chiral centers. This resulted in 283 compounds for in silico screening. We generated ~15,200 energetically favorable conformations for in silico screening. We docked these conformations into the GTP binding site of IMPDH2 and ranked them in order based on a scoring function. We selected 100 top-scoring compound-IMPDH2 complexes and selected 38 as the unique compounds based on National Service Center (NSC) identity. We obtained 15 compounds from the National Cancer Institute (NCI) and tested them for enzyme activity inhibition against recombinant human IMPDH2 in 96-well plate screening.

### 2.9. Cell Proliferation Assay

For the cell proliferation assay, the aforementioned cell lines were plated in 96-well plates at a density of 20,000 to 40,000 cells per well in standard RPMI medium, with DMSO as a control or with the respective drug concentrations in 100 µL of medium. After 48 h in culture, cell proliferation was assessed using the WST-1 or MTT assay method following the manufacturer’s protocol.

### 2.10. Colony Formation Assay

The colony formation assay used Human Methylcellulose Complete Media (Catalog #: HSC003, R&D Systems, Minneapolis, MN, USA). In brief, 200 cells per well in triplicate (in a 6-well plate) were placed in Human Methylcellulose media according to the manufacturer’s protocol, with a DMSO control, comp-10 (100 nM), and MPA (100 nM). After plating the cells, the plate was incubated in a standard cell culture chamber for four weeks. Colony formation was then visualized using the iBright imaging system, 1500 (Thermo-Fisher Inc. Waltham, MA, USA), and the number of cells was counted.

### 2.11. Statistics and Reproducibility

The Student’s *t*-test was used to analyze differences in Western blot densitometric values and activity assays to assess differences in cell growth and colony formation. *p*-values equal to or less than 0.05 were considered statistically significant without being adjusted for multiple comparisons. The statistical analysis was performed using GraphPad Prism 7.0. software and NIH ImageJ 2 software for the densitometric quantitation of Western blot data.

### 2.12. Data Availability

We confirm that all relevant data and methods are included in the main Article and the Supplementary Information section.

## 3. Results

### 3.1. IMPDH2 Is Significantly Overexpressed in Hematological Malignancies

IMPDH1 and IMPDH2 are rate-limiting enzymes in purine biosynthesis, essential during rapid cell growth. IMPDH1 is constantly expressed, while IMPDH2 is inducible and frequently overexpressed in cancer. The isoforms share about 85% sequence similarity (Figure S1, in the Supplementary Materials). RNA-seq data from the Human Protein Atlas reveal widespread IMPDH2 overexpression compared to IMPDH1 across many cancer cell lines (Figure S2a,b, in the Supplementary Materials). In hematological cancers like mantle cell lymphoma (MCL), diffuse large B-cell lymphoma (DLBCL), chronic lymphocytic leukemia (CLL), acute myeloid leukemia (AML), and anaplastic large cell lymphoma (ALCL), IMPDH2 levels are significantly higher than IMPDH1 (Figure S2c, in the Supplementary Materials). Likewise, leukemia and multiple myeloma cell lines mainly express IMPDH2 with little IMPDH1 (Figure S2d–g, in the Supplementary Materials). These findings emphasize IMPDH2’s upregulation in cancer and support its potential as a therapeutic target.

### 3.2. In Vitro Kinase and Phosphoproteomic Analyses Reveal Tyrosine Phosphorylation of IMPDH2 in the Allosteric Domain

**Figure 1:**
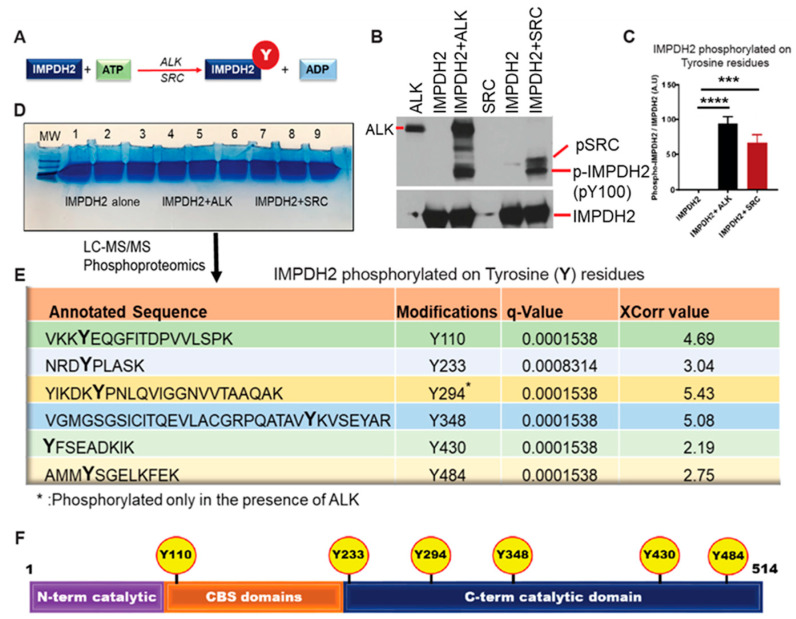
Oncogenic kinases ALK and SRC phosphorylate IMPDH2 at specific tyrosine residues. (**A**), Schematic of the in vitro kinase assay used to evaluate tyrosine phosphorylation of IMPDH2 by ALK and SRC kinases. (**B**), Western blot analysis of in vitro kinase reactions using ALK and SRC with recombinant IMPDH2, probed with phospho-tyrosine-specific and total IMPDH2 antibodies. (**C**), Densitometric quantification of tyrosine-phosphorylated IMPDH2 compared to total IMPDH2 levels, demonstrating enhanced phosphorylation in the presence of ALK and SRC. We compared pY peptides of IMPDH2 alone or in the presence of IMPDH2 in ALK or SRC. (**D**), Large-scale in vitro kinase assay products were resolved by NuPAGE and analyzed by LC-MS/MS phosphoproteomics to identify phosphorylation sites. (**E**), Identification of key tyrosine residues on IMPDH2 phosphorylated by ALK and SRC, as determined by mass spectrometry. (**F**), Schematic of IMPDH2 structural domains highlighting novel ALK- and SRC-mediated phosphorylation sites. All graphs represent mean ± SD from three replicates (*n* = 3). Statistical significance was assessed using unpaired *t*-tests or one-way ANOVA: *** *p* < 0.001, **** *p* < 0.0001.

IMPDH1 and IMPDH2 are highly expressed in proliferative lymphoid malignancies, including T- and B-cell lymphomas. This study focuses on two aggressive subtypes: ALK-positive ALCL and MCL. Given the role of tyrosine phosphorylation in regulating metabolic enzymes in tyrosine kinase-driven cancers, we investigated whether oncogenic kinases phosphorylate IMPDH2. While databases like the Human Protein Atlas and PhosphoSitePlus report IMPDH2 expression and candidate phospho-tyrosine sites, functional evidence is lacking. In vitro kinase assays with recombinant IMPDH2 and active ALK or SRC kinases revealed robust tyrosine phosphorylation, confirmed by immunoblotting (Figure 1A𢀓C). Mass spectrometry identified SRC-targeted sites Y110, Y233, Y348, Y430, and Y484, while ALK shared four and uniquely phosphorylated Y294 (Figure 1D,E). Notably, Y110 and Y233 map to the allosteric CBS domains, suggesting a regulatory function. Sequence alignment showed that Y110 is replaced by F110 in IMPDH1, highlighting isoform-specific divergence (Figure S3a,b, in the Supplementary Materials). A schematic of phosphorylation sites is shown in Figure 1F.

### 3.3. IMPDH2 Y233 Phosphorylation Regulates PI3P Binding

**Figure 2:**
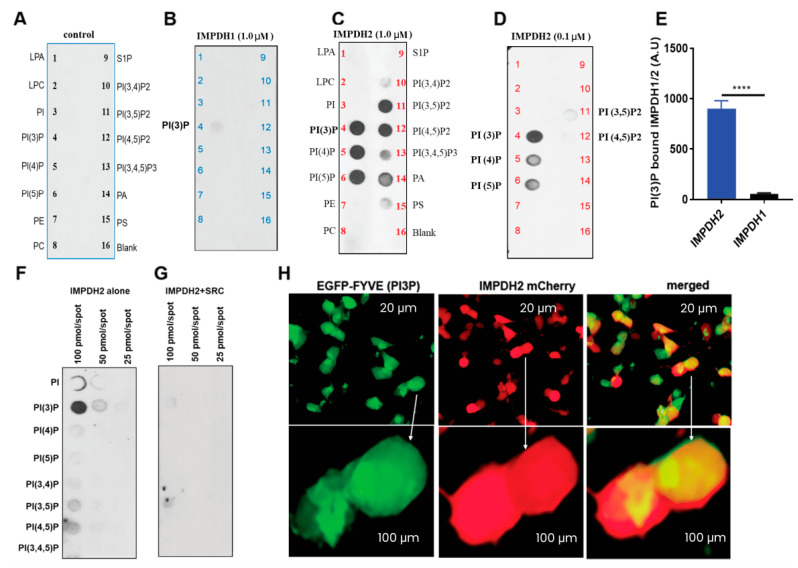
**IMPDH2 Y233 phosphorylation regulates PI3P binding**. (**A**), Schematic of PIP-lipid strip assay showing membranes coated with 15 distinct phospholipid species and incubated with HA peptide as a negative control. (**B**), Recombinant human IMPDH1 (1.0 µg) incubated on PIP-lipid strips in 3% BSA/PBS overnight at 4 °C; binding detected by Western blot using IMPDH1 antibody. (**C**), Recombinant human IMPDH2 (1.0 µg) incubated under identical conditions and probed with IMPDH2 antibody. (**D**), Control binding experiment using a reduced amount (0.1 µg) of IMPDH1 and probing with IMPDH2 antibody to assess specificity. (**E**), Densitometric quantification of PI3P binding to IMPDH2 and IMPDH1, demonstrating preferential binding of PI3P to IMPDH2. (**F**,**G**), HA-tagged human IMPDH2 constructs expressed in HEK293T cells, with or without co-expression of SRC kinase; PI3P-bound IMPDH2-HA was detected by Western blot using anti-HA antibody. (**H**), Live-cell imaging of HEK293T cells co-transfected with IMPDH2-mCherry and EGFP-FYVE (PI3P biosensor), revealing co-localization of IMPDH2 with PI3P (Scale bar: 20 μm and 100 μm). All graphs represent mean ± SD from three biological replicates (*n* = 3). Statistical significance was assessed using unpaired *t*-tests: **** *p* < 0.0001.

Sequence differences in lysine and arginine residues between IMPDH1 and IMPDH2 suggest distinct phospholipid binding profiles. To explore this, we used PIP strip membranes to compare phosphoinositide binding. While IMPDH1 bound weakly to PI3P, IMPDH2 showed strong binding to PI3P, PI4P, and phosphatidic acid (Figure 2A–C). IMPDH2 retained strong PI3P affinity even at lower concentrations (Figure 2D,E), and PIP array assays confirmed a concentration-dependent binding to PI3P (Figure 2F,G). Based on our phosphoproteomics data, we hypothesized that Y233 phosphorylation regulates this interaction. In HEK-293T cells, co-expression of IMPDH2 with active SRC kinase significantly reduced PI3P binding (Figure 2G). Additionally, microscopy of cells co-expressing IMPDH2-mCherry and the PI3P marker EGFP-FYVE showed strong co-localization, confirming membrane association (Figure 2H). These findings suggest that Y233 phosphorylation negatively regulates IMPDH2–PI3P interaction.

### 3.4. PI3P Binding Inhibits IMPDH2 Activity

**Figure 3:**
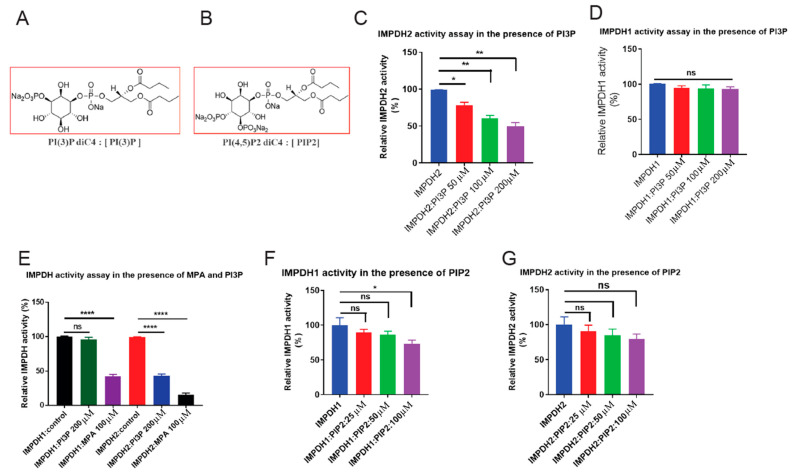
**PI3P binding inhibits IMPDH2 enzymatic activity.** (**A**,**B**), Chemical structures of phospholipids PI3P and PIP2 used in this study. (**C**), Recombinant IMPDH2 was pre-incubated with increasing concentrations of synthetic PI3P; enzymatic activity was measured and showed dose-dependent inhibition. (**D**), Recombinant IMPDH1 was similarly pre-incubated with PI3P to assess its sensitivity to PI3P-mediated inhibition. (**E**), Comparative enzymatic activity of recombinant IMPDH1 and IMPDH2 pre-incubated with 200 µg synthetic PI3P or MPA (mycophenolic acid, a known IMPDH inhibitor) as a positive control. (**F**), Recombinant IMPDH1 was pre-incubated with increasing concentrations of synthetic PIP2; no significant inhibition of enzymatic activity was observed. (**G**), Recombinant IMPDH2 pre-incubated with synthetic PI3P confirms reproducible, dose-dependent inhibition of enzymatic activity. All graphs display mean ± SD from three biological replicates (*n* = 3). Statistical significance was calculated using unpaired *t*-tests: * *p* < 0.05, ** *p* < 0.01, **** *p* < 0.0001. ns is not significant.

Phospholipid–protein interactions often modulate enzymatic activity. To test this, we assessed IMPDH2 activity in the presence of synthetic PI3P and PIP2 (Figure 3A,B). PI3P caused a dose-dependent inhibition of IMPDH2, reducing activity by over 50% at 200 µM (*p* < 0.001) (Figure 3C). At the same time, IMPDH1 remained unaffected (Figure 3D). As a control, mycophenolic acid (MPA) inhibited both isoforms, confirming enzyme responsiveness (Figure 3E). PIP2 did not affect either isoform (Figure 3F,G). These results demonstrate that PI3P selectively and dose-dependently inhibits IMPDH2, but not IMPDH1.

### 3.5. Structure-Based in Silico Screening and Discovery of a Novel Allosteric Inhibitor Targeting IMPDH2

**Figure 4:**
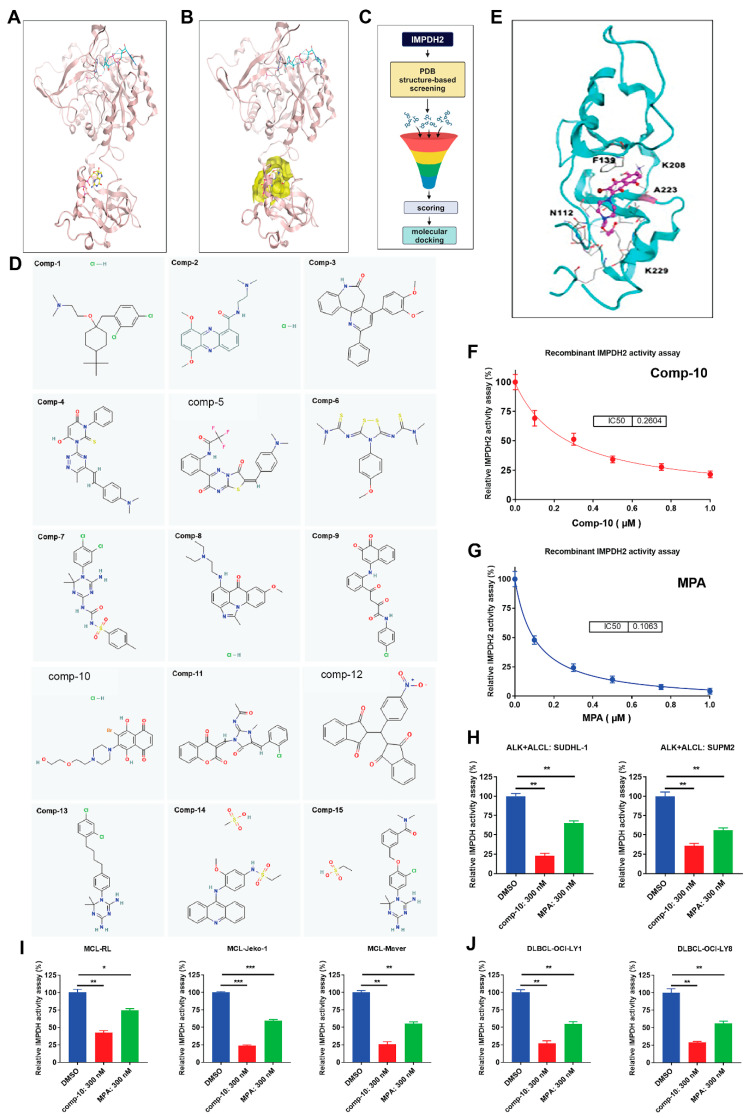
**Structure-based virtual screening identifies a novel small-molecule inhibitor of human IMPDH2**. (**A**), Ribbon diagram of the CBS (cystathionine β-synthase) domain of human inosine monophosphate dehydrogenase 2 (IMPDH2), illustrating the secondary structural elements. (**B**), Surface representation of the GTP-binding pocket (highlighted in yellow) within the IMPDH2 structure, showing the targeted allosteric site for compound docking. (**C**), Schematic overview of the in silico screening pipeline, including structure-based virtual screening, docking, and filtering steps leading to hit identification. (**D**), Chemical structures of 15 top-ranked compounds identified from virtual screening; compound 10 (comp-10) was selected for further validation based on docking scores and predicted binding interactions. (**E**), Molecular docking pose of comp-10 within the GTP-binding pocket of human IMPDH2 (based on the PDB reference structure), revealing key interactions with the binding site residues. (**F**), In vitro enzymatic activity assay of recombinant IMPDH2 in the presence of comp-10, showing concentration-dependent inhibition and determination of the half-maximal inhibitory concentration (IC_50_). (**G**), Enzymatic assay using the clinically established IMPDH inhibitor mycophenolic acid (MPA) as a positive control for benchmarking IC_50_ and assay reproducibility. (**H**–**J**), Inhibition of endogenous IMPDH activity by comp-10 in human lymphoma cell lines: (**H**), anaplastic large cell lymphoma (ALCL); (**I**), mantle cell lymphoma (MCL); and (**J**), diffuse large B-cell lymphoma (DLBCL). Comp-10 treatment significantly reduced IMPDH enzymatic activity across all tested cell lines. All the graphs show mean ± SD (*n* = 3 biological replicates), and all statistical analyses were conducted with unpaired *t*-tests: * *p* < 0.05, ** *p* < 0.01, *** *p* < 0.001.

Building on our findings that IMPDH2 Y233 phosphorylation, PI3P binding, and GTP all regulate IMPDH2 activity via the allosteric domain, we identified lead compound-10 as a novel and selective IMPDH2 inhibitor. For in silico screening, we used the published PDB structure of the IMPDH2 allosteric GTP-binding domain [29] as shown (Figure 4A,B). We performed in silico screening using the National Cancer Institute (NCI) Mechanistic Set VI, comprising 811 compounds selected from 37,836 based on diverse growth inhibition profiles in the NCI-60 cell line screen. Compounds were clustered using the FASTCLUS algorithm in SAS, yielding 1272 clusters, from which representative compounds were selected. After applying filters (MW 100–500, single molecule, 2–6 rotatable bonds, Lipinski drug-like = 1, no chiral centers), 283 compounds remained. Approximately 15,200 energetically favorable conformations were generated and docked into the GTP-binding site of IMPDH2. The top 100 compound-IMPDH2 complexes were ranked, and 38 unique compounds were identified based on NSC identity (Figure 4C). We obtained 15 compounds from the NCI, and their 2D structures are shown (Figure 4D). From these, 25 structural analogs were also identified (Figure S4, in the Supplementary Materials). Initial enzyme activity screening at 10 µM identified three active compounds: comp-5 (>32% inhibition), comp-10 (>99%), and comp-12 (>52%). At 1 µM, comp-10 showed the most potent inhibition (>93%) of recombinant human IMPDH2 activity. Docking analysis of comp-10 with IMPDH2 is shown (Figure 4E). Dose–response assays comparing comp-10 and mycophenolic acid (MPA) yielded IC_50_ values of ~260 nM and ~106 nM, respectively (Figure 4F,G).

Additionally, in silico ADME profiling of Comp-10 was performed using the SwissADME web tool to assess its pharmacokinetics, drug-likeness, and medicinal chemistry friendliness, as described previously [30]. To compare ADME (Absorption, Distribution, Metabolism, and Excretion) properties, we included mycophenolic acid (MPA), a known inhibitor of IMPDH, as a positive control. SwissADME analysis showed that Comp-10 exhibited similar ADME properties to MPA across all pharmacokinetic and drug-likeness parameters (Figure S5a,b, in the Supplementary Materials) and fully adhered to Lipinski’s Rule of Five. Based on the Comp-10 structure, we have also identified several analogs that are currently being tested for IMPDH inhibition and cell growth suppression. To assess cellular IMPDH1/2 inhibition, we treated ALCL (SUDH-L1, SUPM2) and MCL (RL, Jeko-1, Maver) cell lines with 300 nM Comp-10 for 24 h. IMPDH activity assays using equal amounts of DMSO- and Comp-10-treated lysates (5–10 µg per well, *n* = 3) revealed strong inhibition in SUDH-L1 (>77%; *p* < 0.001) and SUPM2 (>64%; *p* < 0.001), compared to modest inhibition by MPA (36% and 44%, respectively; Figure 4H). Similarly, Comp-10 significantly reduced IMPDH activity in MCL lines: RL (>58%; *p* < 0.001), Jeko-1 (>74%; *p* < 0.0001), and Maver (>75%; *p* < 0.0001) (Figure 4I). In DLBCL cell lines OCI-LY1 and OCI-LY8, Comp-10 led to robust inhibition exceeding 73% and 71%, respectively (*p* < 0.001) (Figure 4J).

### 3.6. Comp-10 Downregulates IMPDH1/2 and Prevent Rod/Ring Formation

**Figure 5:**
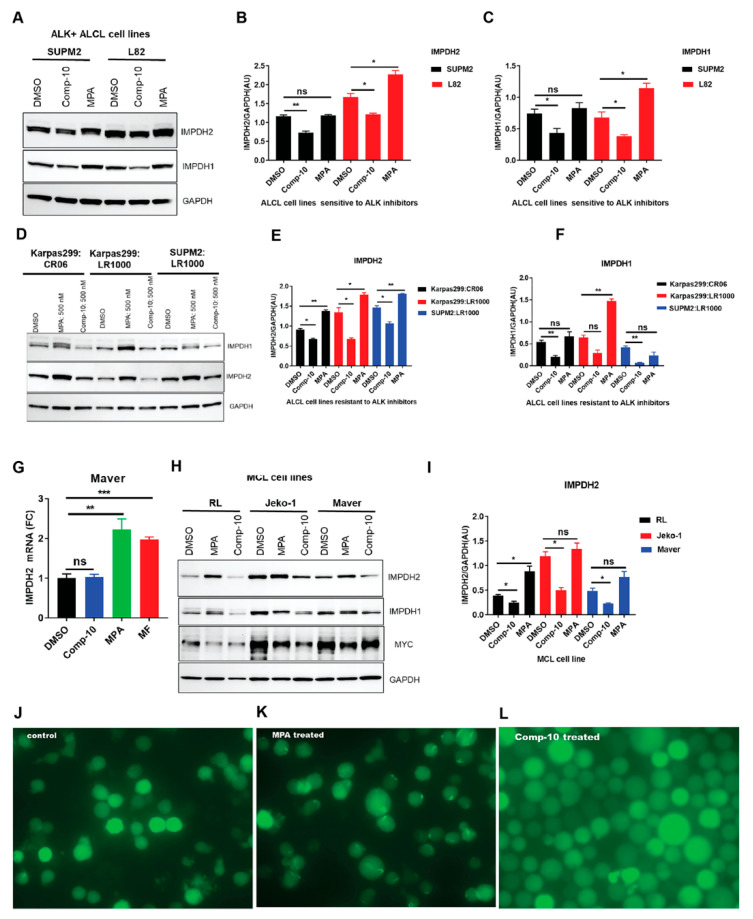
**Comp-10 downregulates IMPDH1/2 expression and prevents filament (rod/ring) formation in ALCL and MCL models.** (**A**), Western blot analysis of IMPDH1 and IMPDH2 expression in ALK inhibitor (ALKi)-sensitive ALCL cell lines (SUPM2 and L82) following 48-h treatment with Comp-10 (300 nM) or mycophenolic acid (MPA). (**B**,**C**), Densitometric quantification of IMPDH1 and IMPDH2 protein bands from panel A, confirming significant downregulation following Comp-10 treatment. (**D**), Western blot of ALKi-resistant ALCL cell lines (Karpas299 and SUPM2-CR) treated with Comp-10 and MPA, showing consistent downregulation of IMPDH1/2 by Comp-10 and upregulation by MPA. (**E**,**F**), Densitometric analysis of protein expression in resistant ALCL lines (panel D) supports differential regulation by Comp-10 and MPA. (**G**), Quantitative RT–PCR analysis of IMPDH2 mRNA expression in MCL cell line Maver treated with Comp-10, MPA and MF. (**H**), Western blot analysis of BTK inhibitor (BTKi)-sensitive and -resistant MCL cell lines treated with Comp-10 and MPA. (**I**), Densitometric quantification of Western blots shown in panel H, confirming significant downregulation of IMPDH1/2 protein levels by Comp-10 across multiple MCL models. (**J**–**L**), Light microscopy of SUDHL-1 cells expressing HA–GFP–IMPDH2 shows distinct patterns of filament formation: DMSO-treated control cells. (**J**), lack filaments; MPA-treated cells. (**K**), display prominent rod/ring structures; and Comp-10-treated cells. (**L**), show no rod/ring or filament formation (scale bar: 20 μm). Densitometric values of the Western blots (*n* = 2). All the graphs show mean ± SD (*n* = 3 biological replicates), and all statistical analyses were conducted with unpaired *t*-tests: * *p* < 0.05, ** *p* < 0.01, *** *p* < 0.001; ns is not significant.

We compared the effects of Comp-10 and mycophenolic acid (MPA) on IMPDH1 and IMPDH2 expression in ALK-positive ALCL and MCL cell lines, including models sensitive and resistant to ALK inhibitors (ALKis) crizotinib and lorlatinib. In ALKi-sensitive SUPM2 and L82 cells, treatment with Comp-10 (300 nM, 48 h) markedly reduced IMPDH1/2 protein levels, while MPA modestly increased their expression (Figure 5A–C). Similar trends were observed in resistant lines (Karpas299 and SUPM2-CR), with Comp-10 downregulating and MPA upregulating IMPDH1/2 (Figure 5D–F). In MCL cells (Maver), qRT-PCR revealed that Comp-10 did not induce IMPDH2 transcription, while both MPA and mizoribine (MF) significantly upregulated it (Figure 5G). Across BTKi-sensitive and -resistant MCL lines, Comp-10 consistently reduced IMPDH1/2 protein levels, whereas MPA increased them. MYC expression remained unchanged, suggesting Comp-10 acts post-transcriptionally (Figure 5H). Densitometry confirmed significant protein reductions with Comp-10 (Figure 5I). We also assessed IMPDH filament (rod/ring) formation in SUDHL-1 cells expressing HA-GFP–tagged IMPDH2. DMSO-treated controls showed no filaments (Figure 5J), MPA induced prominent rods/rings (Figure 5K), while Comp-10 prevented filament formation (Figure 5L).

In summary, IMPDH2 is overexpressed relative to IMPDH1 in ALCL and MCL. MPA, a catalytic-site inhibitor, upregulates IMPDH1/2 and induces rods/rings, possibly contributing to cytotoxicity. In contrast, Comp-10 targets the regulatory domain, suppresses IMPDH1/2 post-transcriptionally, blocks filament formation, and retains efficacy in drug-resistant models, representing a mechanistically distinct and potentially superior IMPDH inhibitor.

### 3.7. Comp-10 Inhibits Growth and Colony Formation of ALK-Positive Malignant Cells

**Figure 6:**
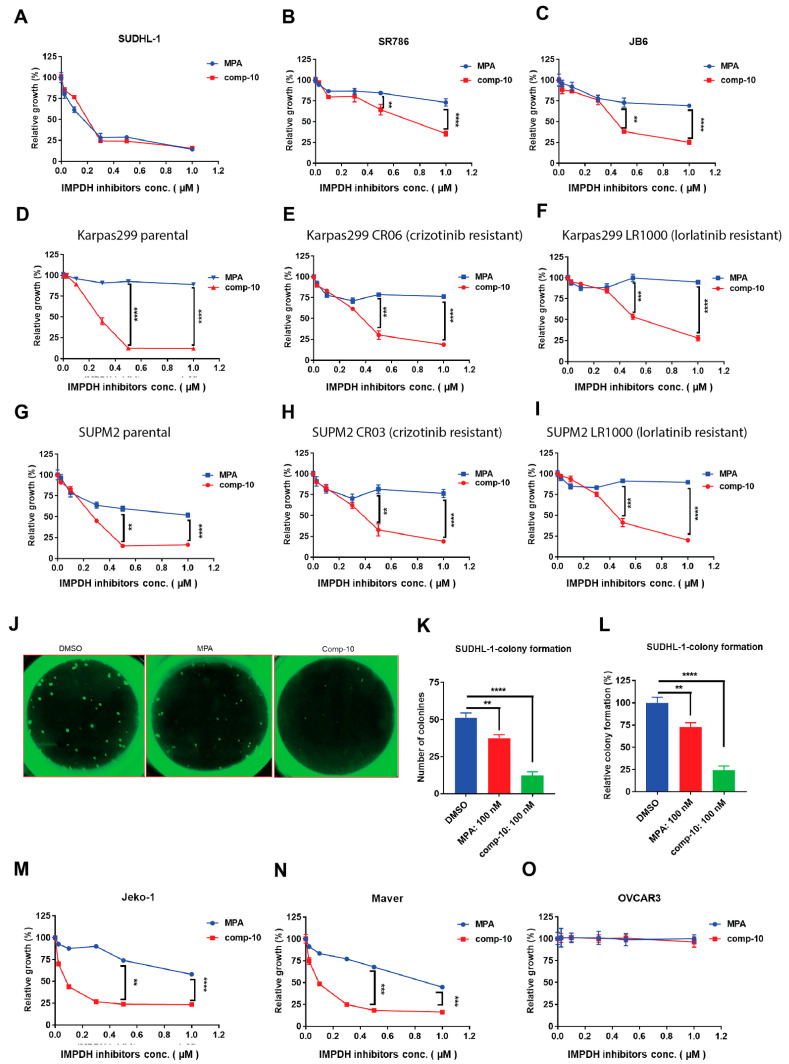
Comp-10 inhibits proliferation and colony formation in ALK-positive lymphoma cells. (**A**–**C**), Cell viability assays of ALCL cell lines treated with comp-10 or mycophenolic acid (MPA). While both compounds similarly inhibited growth in one line. (**A**) comp-10 showed significantly stronger growth inhibition in two additional lines. (**B**–**F**), Comp-10 potently suppressed the growth of ALK inhibitor (ALKi)-sensitive Karpas 299 cells (**D**) and their resistant derivatives: Crizotinib-resistant (**E**) and Lorlatinib-resistant (**F**) lines. (**G**–**I**), Similar results were observed in parental SUP-M2 cells (**G**) and their ALKi-resistant derivatives (**H**,**I**), with comp-10 maintaining robust antiproliferative activity. (**J**), Representative GFP fluorescence images of colonies formed by SUDHL-1 cells stably expressing IMPDH2–HA–GFP in methylcellulose after 4 weeks of treatment with DMSO, comp-10 (100 nM), or MPA (100 nM). (**K**), Quantification of colony numbers per well. Comp-10 significantly reduced colony formation compared to both DMSO (*p* < 0.0001) and MPA (*p* < 0.01). (**L**), Colony numbers expressed as percentage of DMSO control. Comp-10 reduced colony formation to ~12%, while MPA-treated cells retained > 72% colony formation relative to control. (**M**,**N**), Cell viability assays in two mantle cell lymphoma (MCL) index lines demonstrate that comp-10 effectively inhibits cell growth than MPA. (**O**), Growth of the ovarian carcinoma cell line OVCAR3 remained unaffected by comp-10 or MPA, indicating minimal off-target toxicity in non-lymphoid malignancies. All the graphs show mean ± SD (*n* = 3 biological replicates), and all statistical analyses were conducted with unpaired *t*-tests: ** *p* < 0.01, *** *p* < 0.001, **** *p* < 0.0001.

To assess the anti-proliferative effects of Comp-10, we tested ALK-positive ALCL cell lines using MPA as a positive control. Comp-10 showed equal or greater growth inhibition than MPA across multiple lines. In particular, it was more effective in two of three tested lines (Figure 6A–C). Notably, Comp-10 suppressed the growth of both ALK inhibitor (ALKi)-sensitive parental Karpas 299 cells (Figure 6D) and their crizotinib- and lorlatinib-resistant cells (Figure 6E,F). Similar results were observed in SUP-M2 cells and their resistant counterparts (Figure 6G–I). We next evaluated the effect of Comp-10 on colony formation using SUDHL-1 cells stably expressing IMPDH2-HA-GFP. Cells were plated in methylcellulose and treated with DMSO, Comp-10 (100 nM), or MPA (100 nM). After 4 weeks, colony counts revealed a significant reduction in Comp-10–treated wells (12% of DMSO control, *p* < 0.0001), while MPA-treated cells retained 72% colony formation (*p* < 0.002) (Figure 6J–L). In MCL index cell lines, Comp-10 also outperformed MPA in suppressing cell proliferation (Figure 6M,N). In contrast, the ovarian carcinoma cell line OVCAR3 was unaffected by either drug (Figure 6O), suggesting minimal off-target toxicity.

**Figure 7:**
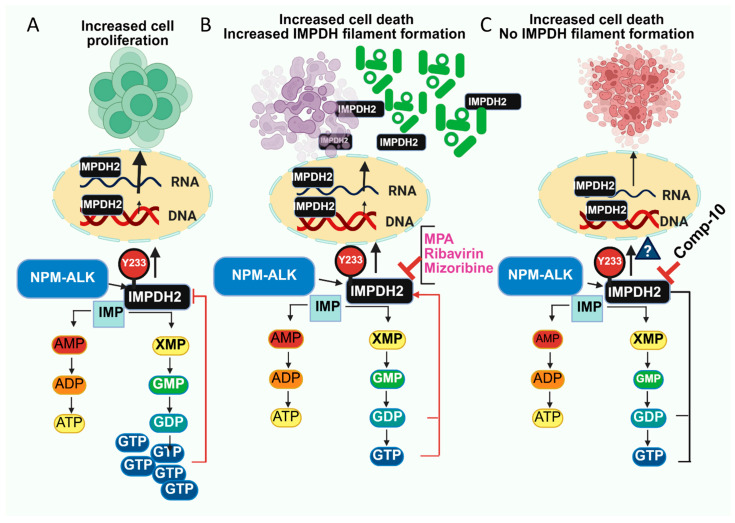
Schematic representation of IMPDH2 regulation and inhibition mechanisms. (**A**), In normal cells, IMPDH2 catalyzes the conversion of inosine monophosphate (IMP) to xanthosine monophosphate (XMP), a critical step in GTP biosynthesis. Elevated GTP levels act as a negative feedback regulator of IMPDH2 activity, maintaining cellular homeostasis and supporting proliferation. (**B**) Treatment with classical IMPDH inhibitors (e.g., MPA, ribavirin, or mizoribine) blocks IMPDH1/2 enzymatic activity, depleting intracellular GTP levels. This loss of feedback inhibition triggers compensatory overexpression of IMPDH1/2 and the formation of cytoplasmic rods and rings (RR) structures. (**C**), In contrast, the novel allosteric inhibitor Comp-10 targets the regulatory domain of IMPDH2, reducing both its enzymatic activity and protein abundance without inducing RR formation. This post-transcriptional mechanism represents a distinct and potentially less toxic approach to inhibiting IMPDH2.

Based on these findings, we summarize our results schematically in Figure 7. In normal cells, IMPDH2 regulates the biosynthesis of GTP. It supports cell proliferation, with GTP acting as a negative feedback regulator of IMPDH activity (Figure 7A). When treated with classical IMPDH1/2 inhibitors such as MPA, Ribavirin, or Mizoribine, enzymatic activity is blocked, leading to decreased GTP levels. This interrupts the feedback loop and causes compensatory overexpression of IMPDH1/2, often accompanied by rods and rings (RR) filament formation (Figure 7B). In contrast, treatment with the novel allosteric inhibitor Comp-10 reduces IMPDH2 enzymatic activity and protein levels without inducing RR formation. Comp-10 binds to the regulatory domain, offering a distinct, potentially less toxic mechanism of IMPDH2 inhibition that acts post-transcriptionally, providing a different and possibly less toxic way to inhibit IMPDH2 (Figure 7C).

## 4. Discussion

Our study provides the first direct evidence that oncogenic tyrosine kinases (TKs), specifically ALK and SRC, phosphorylate IMPDH2, a key enzyme in purine biosynthesis. Although tyrosine phosphorylation accounts for a small part of total post-translational modifications, it plays a vital role in regulating metabolism, proliferation, and survival. While IMPDH2 overexpression is common in cancers, the functional significance of its phosphorylation has largely remained unexplored. We show that phosphorylation at the conserved Y233 residue within the IMPDH2 allosteric domain controls its enzymatic activity, directly linking TK signaling to metabolic reprogramming in cancer. This has important therapeutic implications, especially given the rise of resistance to FDA-approved ALK inhibitors such as crizotinib, ceritinib, alectinib, brigatinib, and lorlatinib. Notably, crizotinib is also approved for NPM-ALK-positive ALCL and neuroblastoma, but resistance continues to be a major clinical challenge.

Targeting downstream effectors like IMPDH2 could offer an alternative way to overcome resistance. We show that inhibiting IMPDH reduces the growth of both ALK inhibitor-sensitive and -resistant ALCL cells, highlighting IMPDH2 as a promising therapeutic target. Since ALK (EML4-ALK) and SRC play a role in other cancers, this approach may have broader relevance. We also identify PI3P as a natural lipid regulator of IMPDH2, binding specifically to basic residues within the CBS domains and inhibiting its enzymatic activity. Y233, found in this regulatory domain, is different from the active-site Cys-331 targeted by traditional inhibitors like mycophenolic acid (MPA). These results emphasize the potential of allosteric modulation as a new approach for inhibiting IMPDH2.

The immunosuppressive potential of IMPDH2 inhibitors, such as MPA and MMF, is well documented and an important consideration in cancer therapy. While Comp-10 inhibits IMPDH2 activity, disrupts R/R formation, and prevents tumor cell proliferation, its effects on the immune system, particularly on anti-tumor immune responses, are not yet fully understood. Although we have not conducted specific studies on Comp-10’s impact on immune cell populations or functions, its structural and functional differences from classical IMPDH2 inhibitors may indicate a unique immunological profile. Nevertheless, we acknowledge that any future translational development of Comp-10 will require careful evaluation of its effects on both tumor-intrinsic and immune-mediated mechanisms.

Using a structure-based computational screening approach, we identified Comp-10, a first-in-class allosteric IMPDH inhibitor. Unlike MPA, which induces filament formation (rods and rings) and paradoxically increases IMPDH protein levels, Comp-10 reduces IMPDH1/2 expression and prevents filament assembly. Comp-10 demonstrated superior efficacy in inhibiting the growth and colony formation of ALK-positive and MCL cells, including drug-resistant models, while sparing non-malignant cells like OVCAR3. The advantages of allosteric inhibitors, including reducing IMPDH1/2 protein levels and rods/ring formation, decrease toxicity and highlight the therapeutic potential of Comp-10 promise. With drug development timelines spanning 10–15 years and costs exceeding $2 billion, computational strategies like ours offer a cost-effective and accelerated path to drug discovery.

## 5. Conclusions

IMPDH2 is overexpressed in hematologic malignancies, including ALCL, MCL, DLBCL, AML, and CLL, where its expression exceeds that of IMPDH1. We demonstrate that tyrosine phosphorylation at Y233 within the CBS domain modulates IMPDH2 activity and its interaction with PI3P, a lipid that selectively inhibits IMPDH2 but not IMPDH1.

Using a structure-guided screen, we identified Comp-10, a first-in-class allosteric inhibitor targeting the regulatory domain of IMPDH2. Comp-10 shows potent and selective activity across multiple hematologic cancer models, surpassing traditional catalytic-site inhibitors like MPA. It suppresses IMPDH1/2 protein levels, avoids filament induction, and spares non-hematologic cells, suggesting a safer and more targeted mechanism. These findings establish IMPDH2 as a mechanistically tractable, cancer-selective metabolic target. Comp-10 represents a promising therapeutic strategy for ALK-driven and BTK inhibitor-resistant lymphomas and potentially other tyrosine kinase-driven malignancies.

## Data Availability

The data presented in this study are openly available in Biorxiv https://doi.org/10.1101/2025.08.19.667179.

## Supplementary Materials

The following supporting information can be downloaded at: https://www.mdpi.com/article/10.3390/cancers17203389/s1, Figure S1: Sequence alignment of human IMPDH1 and IMPDH2; Figure S2: IMPDH2 is broadly overexpressed across human cancers; Figure S3: IMPDH1 F110 and IMPDH2 Y110 CBS-1 domain sequence homology of various species; Figure S4: Structure-based in silico screening and identification of novel IMPDH2 inhibitor analogs; Figure S5: SwissADME analysis predicts favorable drug-likeness and oral bioavailability of Comp-10.
