# Supplementary material for: A Novel Allosteric Inhibitor Targeting IMPDH at Y233 Overcomes Resistance to Tyrosine Kinase Inhibitors in Lymphoma
Source: Cancers (Basel). 2025 Oct 21;17(20):3389. doi: 10.3390/cancers17203389 (PMC12563536; doi:10.3390/cancers17203389)
Supplement: Supplementary file 1 [file cancers-17-03389-s001.zip › cancers-3907123-supplementary.pdf]

Supplementary Fig.S1

Human Inosine-5'-monophosphate dehydrogenase 1 (IMPDH1) and IMPDH2 sequence alignment

```
CLUSTAL O(1.2.4) multiple sequence alignment


NP_001136045.1      MADYLISGGTGYVPEDGLTAQQLFASADGLTYNDFLILPGFIDFIADEVDLTSALTRKIT   60
NP_000875.2         MADYLISGGTSYVPDDGLTAQQLFNCGDGLTYNDFLILPGYIDFTADQVDLTSALTKKIT   60
                    **********.***:********* ..*************:*** **:********:***

                                                                  IMPDH1 F110
NP_001136045.1      LKTPLISSPMDTVTEADMAIAMALMGGIGFIHHNCTPEFQANEVRKVKKFEQGFITDPVV   120
NP_000875.2         LKTPLVSSPMDTVTEAGMAIAMALTGGIGFIHHNCTPEFQANEVRKVKKYEQGFITDPVV   120
                    *****:**********.******* ************************:**********
                                                                  IMPDH2 Y110

NP_001136045.1      LSPSHTVGDVLEAKMRHGFSGIPITETGTMGSKLVGIVTSRDIDFLAEKDHTTLLSEVMT   180
NP_000875.2         LSPKDRVRDVFEAKARHGFCGIPITDTGRMGSRLVGIISSRDIDFLKEEEHDCFLEEIMT   180
                    ***.. * **:*** ****.*****:** ***:****::******* *::*  :*.*:**

                                                                      IMPDH1 Y233
NP_001136045.1      PRIELVVAPAGVTLKEANEILQRSKKGKLPIVNDCDELVAIIARTDLKKNRDYPLASKDS   240
NP_000875.2         KREDLVVAPAGITLKEANEILQRSKKGKLPIVNEDDELVAIIARTDLKKNRDYPLASKDA   240
                     * :*******:*********************: ************************:
                                                                      IMPDH2 Y233

NP_001136045.1      QKQLLCGAAVGTREDDKYRLDLLTQAGVDVIVLDSSQGNSVYQIAMVHYIKQKYPHLQVI   300
NP_000875.2         KKQLLCGAAIGTHEDDKYRLDLLAQAGVDVVVLDSSQGNSIFQINMIKYIKDKYPNLQVI   300
                    :********:**:**********:******:*********::** *::***:***:****

NP_001136045.1      GGNVVTAAQAKNLIDAGVDGLRVGMGCGSICITQEVMACGRPQGTAVYKVAEYARRFGVP   360
NP_000875.2         GGNVVTAAQAKNLIDAGVDALRVGMGSGSICITQEVLACGRPQATAVYKVSEYARRFGVP   360
                    *******************.******.*********:******.******:*********

NP_001136045.1      IIADGGIQTVGHVVKALALGASTVMMGSLLAATTEAPGEYFFSDGVRLKKYRGMGSLDAM   420
NP_000875.2         VIADGGIQNVGHIAKALALGASTVMMGSLLAATTEAPGEYFFSDGIRLKKYRGMGSLDAM   420
                    :*******.***:.*******************************:**************

NP_001136045.1      EKSSSSQKRYFSEGDKVKIAQGVSGSIQDKGSIQKFVPYLIAGIQHGCQDIGARSLSVLR   480
NP_000875.2         DKHLSSQNRYFSEADKIKVAQGVSGAVQDKGSIHKFVPYLIAGIQHSCQDIGAKSLTQVR   480
                    :*  ***:*****.**:*:******::******:************.******:**: :*

NP_001136045.1      SMMYSGELKFEKRTMSAQIEGGVHGLHSYEKRLY   514
NP_000875.2         AMMYSGELKFEKRTSSAQVEGGVHSLHSYEKRLF   514
                    :************* ***:*****.********:
```

Supplementary Fig.S2

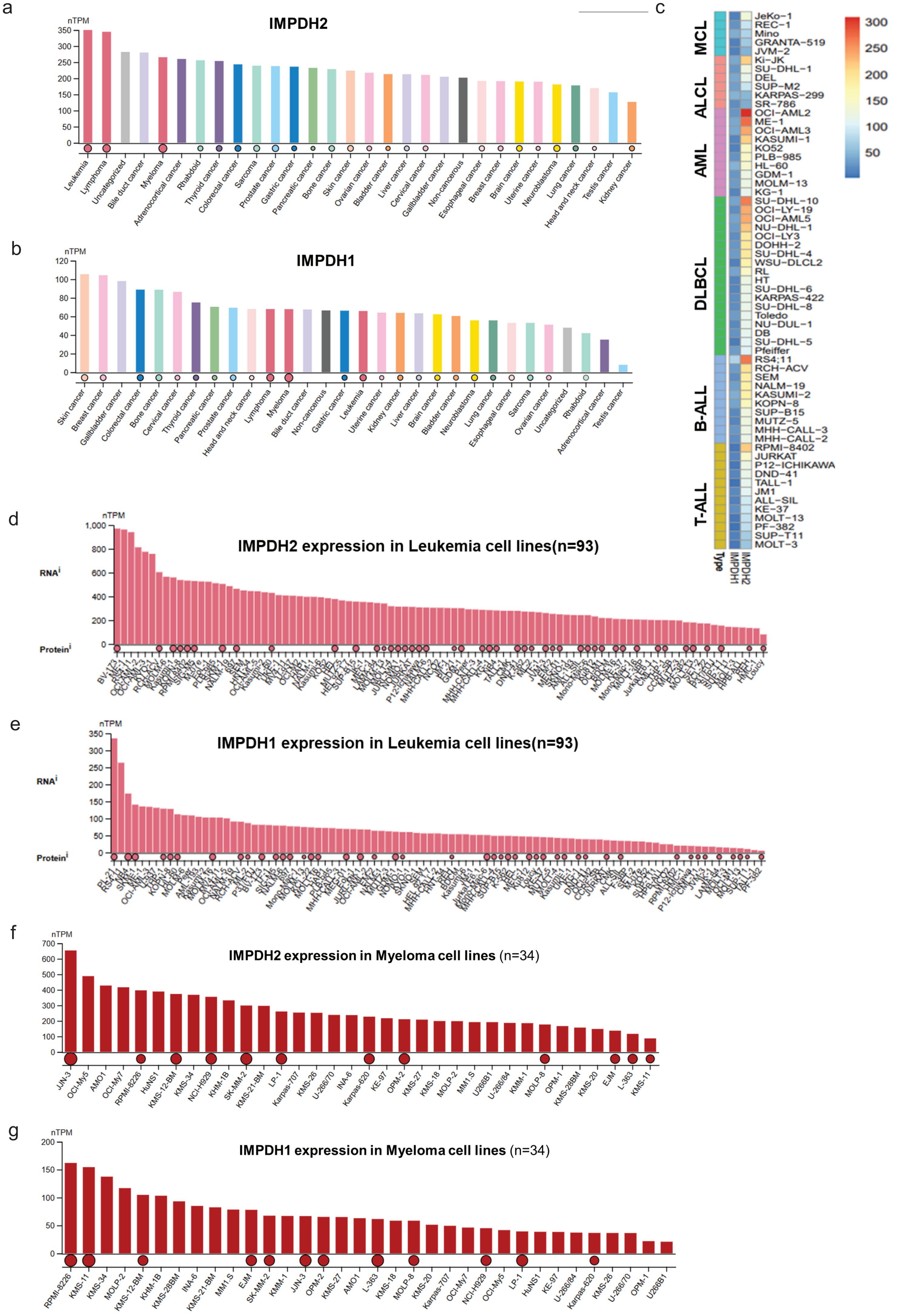

Supplementary Fig.S3

a

IMPDH1										F110							
Species	Scientific Name	P-Site	-7	-6	-5	-4	-3	-2	-1	0	1	2	3	4	5	6	7
Human	Homo sapiens	F110	E	V	R	K	V	K	K	F	E	Q	G	F	I	T	D
Rhesus Macaque	Macaca mulatta	F110	E	V	R	K	V	K	K	F	E	Q	G	F	I	T	D
Dog	Lupus familis	F110	E	V	R	K	V	K	K	F	E	Q	G	F	I	T	D
Mouse	Mus musculus	F110	E	V	R	K	V	K	K	F	E	Q	G	F	I	T	D
Rat	Rattus norvegicus	F110	E	V	R	K	V	K	K	F	E	Q	G	F	I	T	D

b

IMPDH2										Y110							
Species	Scientific Name	P-Site	-7	-6	-5	-4	-3	-2	-1	0	1	2	3	4	5	6	7
Human	Homo sapiens	Y110	E	V	R	K	V	K	K	Y	E	Q	G	F	I	T	D
Rhesus Macaque	Macaca mulatta	Y110	E	V	R	K	V	K	K	Y	E	Q	G	F	I	T	D
Dog	Lupus familis	Y110	E	V	R	K	V	K	K	Y	E	Q	G	F	I	T	D
Mouse	Mus musculus	Y110	E	V	R	K	V	K	K	Y	E	Q	G	F	I	T	D
Rat	Rattus norvegicus	Y110	E	V	R	K	V	K	K	Y	E	Q	G	F	I	T	D
Chicken	Gallus gallus	Y110	E	V	R	K	V	K	K	Y	E	Q	G	F	I	T	D
Frog	Xenopus laevis	Y110	E	V	R	K	V	K	K	Y	E	Q	G	F	I	T	D
Zebra Danio	Brachydanio rerio	Y110	E	V	R	K	V	K	R	Y	E	Q	G	F	I	T	D

Supplementary Fig.4

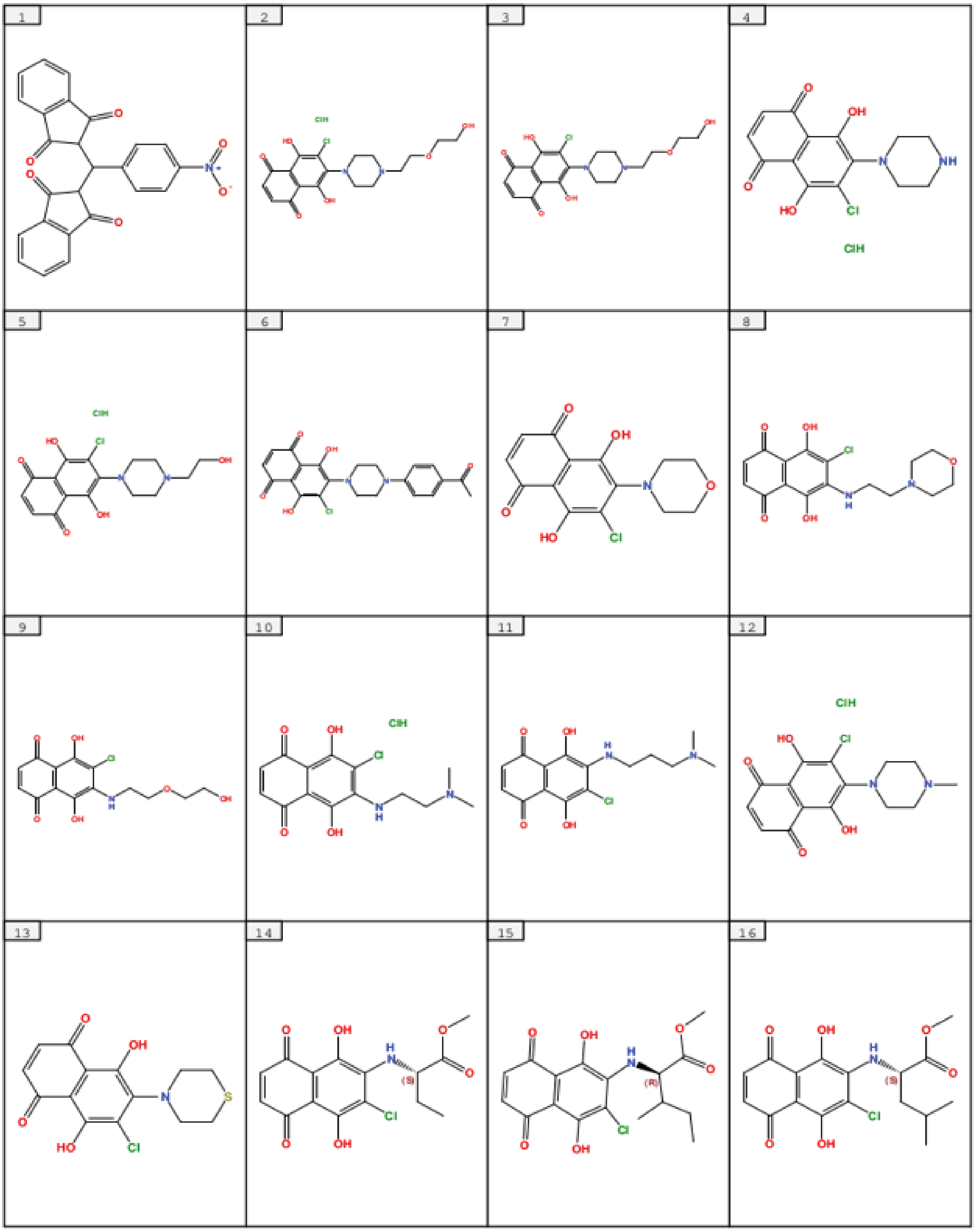

Supplementary Fig.5

a

Comp-10

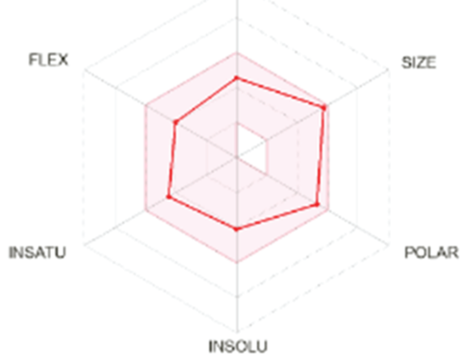

Physicochemical Properties	
Formula	C18H22BrClN2O6
Molecular weight	477.73 g/mol
Num. heavy atoms	28
Num. arom. heavy atoms	6
Fraction Csp3	0.44
Num. rotatable bonds	6
Num. H-bond acceptors	7
Num. H-bond donors	3
Molar Refractivity	115.88
TPSA	110.54 Å²
**Lipophilicity**	
Log $P_{o/w}$ (iLOGP)	0.00
Log $P_{o/w}$ (XLOGP3)	2.44
Log $P_{o/w}$ (WLOGP)	0.97
Log $P_{o/w}$ (MLOGP)	-0.31
Log $P_{o/w}$ (SILICOS-IT)	1.59
Consensus Log $P_{o/w}$	0.94

Water Solubility	
Log *S* (ESOL)	-4.10
Solubility	3.78e-02 mg/ml ; 7.91e-05 mol/l
Class	Moderately soluble
Log *S* (Ali)	-4.40
Solubility	1.88e-02 mg/ml ; 3.94e-05 mol/l
Class	Moderately soluble
Log *S* (SILICOS-IT)	-2.97
Solubility	5.13e-01 mg/ml ; 1.07e-03 mol/l
Class	Soluble
**Pharmacokinetics**	
GI absorption	High
BBB permeant	No
P-gp substrate	Yes
CYP1A2 inhibitor	Yes
CYP2C19 inhibitor	No
CYP2C9 inhibitor	No
CYP2D6 inhibitor	No
CYP3A4 inhibitor	No
Log K_p (skin permeation)	-7.48 cm/s
**Druglikeness**	
Lipinski	Yes; 0 violation
Ghose	Yes
Veber	Yes
Egan	Yes
Muegge	Yes
Bioavailability Score	0.55
**Medicinal Chemistry**	
PAINS	1 alert: quinone_A
Brenk	1 alert: hydroquinone
Leadlikeness	No; 1 violation: MW>350
Synthetic accessibility	3.55

b

MPA

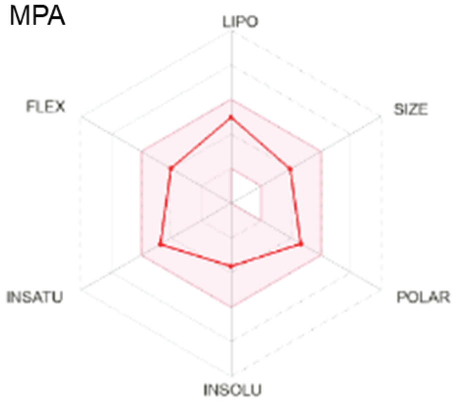

Physicochemical Properties	
Formula	C17H20O6
Molecular weight	320.34 g/mol
Num. heavy atoms	23
Num. arom. heavy atoms	6
Fraction Csp3	0.41
Num. rotatable bonds	6
Num. H-bond acceptors	6
Num. H-bond donors	2
Molar Refractivity	84.35
TPSA	93.06 Å²
**Lipophilicity**	
Log $P_{o/w}$ (iLOGP)	2.48
Log $P_{o/w}$ (XLOGP3)	3.20
Log $P_{o/w}$ (WLOGP)	2.58
Log $P_{o/w}$ (MLOGP)	1.82
Log $P_{o/w}$ (SILICOS-IT)	3.52
Consensus Log $P_{o/w}$	2.72

Water Solubility	
Log *S* (ESOL)	-3.64
Solubility	7.35e-02 mg/ml ; 2.30e-04 mol/l
Class	Soluble
Log *S* (Ali)	-4.83
Solubility	4.78e-03 mg/ml ; 1.49e-05 mol/l
Class	Moderately soluble
Log *S* (SILICOS-IT)	-3.56
Solubility	8.78e-02 mg/ml ; 2.74e-04 mol/l
Class	Soluble
**Pharmacokinetics**	
GI absorption	High
BBB permeant	No
P-gp substrate	No
CYP1A2 inhibitor	Yes
CYP2C19 inhibitor	No
CYP2C9 inhibitor	No
CYP2D6 inhibitor	No
CYP3A4 inhibitor	No
Log K_p (skin permeation)	-5.98 cm/s
**Druglikeness**	
Lipinski	Yes; 0 violation
Ghose	Yes
Veber	Yes
Egan	Yes
Muegge	Yes
Bioavailability Score	0.56
**Medicinal Chemistry**	
PAINS	0 alert
Brenk	1 alert: isolated_alkene
Leadlikeness	Yes
Synthetic accessibility	3.02
